# The Herbal Formula JI017 Induces ER Stress via Nox4 in Breast Cancer Cells

**DOI:** 10.3390/antiox10121881

**Published:** 2021-11-25

**Authors:** Tae Woo Kim, Seong-Gyu Ko

**Affiliations:** Department of Preventive Medicine, College of Korean Medicine, Kyung Hee University, 1 Hoegi, Seoul 130-701, Korea; tae1410@naver.com

**Keywords:** JI017, ER stress, ROS, Nox4, exosome

## Abstract

Chemotherapy is a powerful anti-tumor therapeutic strategy; however, resistance to treatment remains a serious concern. To overcome chemoresistance, combination therapy with anticancer drugs is a potential strategy. We developed a novel herbal extract, JI017, with lower toxicity and lesser side effects. JI017 induced programmed cell death and excessive unfolded protein response through the release of intracellular reactive oxygen species (ROS) and calcium in breast cancer cells. JI017 treatment increased the expression of endoplasmic reticulum (ER) stress markers, including p-PERK, p-eIF2α, ATF4, and CHOP, via the activation of both exosomal GRP78 and cell lysate GRP78. The ROS inhibitors diphenyleneiodonium and N-acetyl cysteine suppressed apoptosis and excessive ER stress by inhibiting Nox4 in JI017-treated breast cancer cells. Furthermore, in paclitaxel-resistant breast cancer cell lines, MCF-7R and MDA-MB-231R, a combination of JI017 and paclitaxel overcame paclitaxel resistance by blocking epithelial-mesenchymal transition (EMT) processes, such as the downregulation of E-cadherin expression and the upregulation of HIF-1α, vimentin, Snail, and Slug expression. These findings suggested that JI017 exerts a powerful anti-cancer effect in breast cancer and a combination therapy of JI017 and paclitaxel may be a potential cancer therapy for paclitaxel resistant breast cancer.

## 1. Introduction

Breast cancer is a leading cause of cancer-related deaths in women worldwide [1]. Breast cancer is often divided in non-invasive and invasive breast cancer, and invasive breast cancer has several subtypes [2]. Non-invasive breast cancer (ductal carcinoma), a rare type of breast cancer, is found in the ducts of the breast and cannot spread outside the breast. Conversely, invasive breast cancer is the most common type and can spread outside the breast. Inflammatory breast cancer is a rare type of invasive breast cancer and makes the skin of the breast look red and feel warm [3]. Triple-negative breast cancer is a subtype of breast cancer that lacks estrogen receptors, progesterone receptors, and human epidermal growth factor receptor 2 (HER2) and spread faster than the other types [4]. Breast cancer is treated using a combination of surgery, chemotherapy, hormone treatment, and radiotherapy [5]. Chemo-resistance is a well-known phenomenon that arises due to many factors, such as epigenetic changes, genetic mutations, and molecular mechanisms [6]. Chemotherapy and radiotherapy, which involves the use of X-rays and gamma rays, are potential tumor therapy strategies; however, the problems of resistance are encountered in many patients. Although chemotherapy and radiotherapy can be effective against many cancer types, including non-small-cell lung cancer, prostate cancer, skin cancer, cervical cancer, and neck and head carcinoma, they are not as efficacious against other cancers, such as bladder cancer, glioblastoma, and breast cancer [7]. Radiation and anti-tumor reagents targeted at killing tumor cells can also kill normal cells in the process; therefore, incorporating herbal medicine as a part of a combination therapy may be a potential strategy to reduce the adverse effects [8]. Paclitaxel (brand name: Taxol), which is found in the bark extract, is a member of the taxane class of anticancer drugs and is the most common tumor therapeutic agent used in various cancer types [9]. However, paclitaxel-resistance is often observed. A recent report suggested that the epithelial-mesenchymal transition (EMT) induces paclitaxel resistance in paclitaxel-treated breast cancer cells [10]. Therefore, combination therapy using paclitaxel and novel anticancer drugs may provide a potential therapeutic strategy for the clinical treatment of breast cancer.

Traditional herbal medicines have long been used for treating cancers, and Korean *Angelica gigas* (Korean Danggui) exerts anticancer, antiangiogenic, and antimetastatic effects in various cancer models [11]. Decursin, a bioactive compound extracted from Angelica gigas, induces apoptosis and inhibits cell proliferation and migration via the PI3K–Akt axis in HeLa cells [12]. In the allograft mice tumor model, decursin medicates apoptosis and blocks invasion through the downregulation of HIF-1α and PD-L1 expression in hypoxia-exposed cancer cells [13]. Decursin also mediates apoptosis through the phosphorylation of JNK and p38 and the downregulation of Bcl-2, CDK4, and cyclin D1 in glioblastoma cells U87 [14]. The ethanol extract of Angelica gigas inhibits cell viability and colony formation and induces apoptosis and G0/G1 arrest via the inhibition of cyclin D1, CDK4, MMP-2, MMP-9, and p38 phosphorylation in the pancreatic cancer cells PANC-1 and PaCa-2 [15]. Traditional Chinese medicine DangguiBuxue Tang (DBT) exerts anti-cancer effects in colorectal cancer cells, and a combination of DBT and radiation has more powerful anticancer effects compared to radiation or DBT alone [16]. Ginger extracts have long been used for treating inflammatory diseases and possess anticancer, antioxidant, and antiangiogenesis properties [17,18,19]. Among the active compound of ginger, 6-gingerol [1-(4′-hydroxy-3′-methoxyphenyl)-5-hydroxy-3-decanone] has been studied for its cytotoxic effects in various cancer cell lines, including breast cancer [20]. Further, 6-gingerol induces intracellular and mitochondrial reactive oxygen species (ROS) release, G0/G1 cell cycle arrest, mitochondrial apoptosis, DNA damage, and EGFR/Src/STAT3 signaling in breast cancer cells, MCF-7 and MDA-MB-231 [21].

We developed and studied a novel herbal medication JI017 with intensive anti-tumor effect in various cancer types. JI017 is a combination of herbal extracts from *Angelica gigas* (Ag), *Zingiber officinale Roscoe* (Zo), and *Aconitum carmichaeli* (Ac) in the ratio of 2:1:1. The anti-cancer effects of Ag, Zo, and Ac have been well reported for many types of cancers, including pancreatic cancer, liver cancer, breast cancer, lung cancer, and oral cancer [22,23,24,25].

Combination therapy using two or more anticancer agents to destroy cancer cells is a potential therapeutic strategy [26]. One of the serious obstacles for effective chemotherapy is acquired chemoresistance caused by numerous factors, such as genes, molecules, and mechanisms. Suggesting novel therapeutic strategies to overcome resistance problems, such as chemoresistance and radioresistance, is still a challenge for scientists and oncologists [27]. Based on this problem, novel strategies to overcome chemoresistance were developed; activator, inhibitor, and co-treatment to regulate chemoresistance-related proteins [28,29]. Chemoresistance induces EMT via the activation of EMT transcription factors, including HI, F1, ZEB1, STAT3, Snail, and Slug, thus promoting tumor metastasis [30].

Endoplasmic reticulum (ER) is an organelle for calcium (Ca^2+^) storage and lipid biosynthesis, and ER stress is induced by various stimulus, such as nutrient starvation, hypoxia, low pH, and free radicals, which mediate cell differentiation and proliferation via the activation of unfolded protein response (UPR) in the tumor environment [31]. However, prolonged and excessive ER stress induces cell death via the activation of UPR sensors, including PERK, IRE1α, and ATF6, and has immunosuppressive effects in the tumor microenvironment [32]. Excessive and prolonged ER stress sensitizes chemoresistant cancer cells, and combination therapy with ER stress inducers mediates apoptosis [33]. Therefore, ER stress may be a potential therapeutic strategy in the tumor microenvironment.

In this study, we investigated the molecular mechanism and anti-cancer effect of JI017 on breast cancer cells in vivo and in vitro. We sought to examine whether JI017 mediates apoptosis via Nox4 expression, ROS and Ca^2+^ production, and the PERK–ATF4–CHOP axis in breast cancer cells. Furthermore, we examined if the effects of the combination of JI017 and paclitaxel can overcome paclitaxel resistance via the inhibition of EMT in paclitaxel-resistant breast cancer cells.

## 2. Materials and Methods

### 2.1. JI017 Extraction

The herbal formula of JI017 was designed as an anticancer drug for breast cancer, and the three ingredients were as follows: Ag, Zo, and a processed Ac. The mixtures were provided by the Jaseng Hospital of Korean medicine (Seoul, Korea). The three ingredients were mixed together in the radio of 2:1:1, soaked in 70% ethanol, and extracted by treatment at 80 °C treatment for 3 h, and then, the extract was filtered, evaporated, and lyophilized to make the JI017 powder. This was stored at −80 °C until use.

### 2.2. Reagents

The following compounds were obtained: diphenyleneiodonium (DPI; Nox and ROS inhibitor, Sigma Aldrich, St. Louis, MO, USA), N-acetylcysteine (NAC; ROS inhibitor, Sigma Aldrich, St. Louis, MO, USA), Z-VAD-FMK (caspase inhibitor, Sigma Aldrich, St. Louis, MO, USA), Paclitaxel (Sigma Aldrich, St. Louis, MO, USA), and thapsigargin (TG; ER stress inducer, Millipore, Bedford, MA, USA).

### 2.3. Cell Cultures

Estrogen receptor-positive MCF-7 and T47D and estrogen receptor-negative SK-BR-3, MDA-MB-231, HCC-1419, and HT20 human breast cancer cell lines were obtained from the Korean Cell Line Bank (Cancer Research Center, Seoul National University, Seoul, Korea). MCF-7, T47D, SK-BR-3, MDA-MB-231, HCC-1419, and HT20 cells were cultured in Dulbecco’s modified Eagle medium (DMEM) (Gibco) supplemented with 10% fetal bovine serum, 100 U/mL penicillin, and 100 mg/mL streptomycin (all from Gibco) and grown at 37 °C under a humidified 95%/5% (*v*/*v*) mixture of air and CO_2_ in an incubator.

### 2.4. Cell Viability

MCF-7 and MDA-MB-231 human breast cancer cell lines were seeded into a 96-well plate with DMEM and grown for 24 h, followed by treatment with JI017 for 24 h. Cell viability assay was performed using the WST-1 assay (Roche, Mannheim, Germany), according to the manufacturer’s protocols. Cell absorbance was measured at 450 nm using an enzyme-linked immunosorbent assay (ELISA) reader (SpectraMax190, Microplate Reader, Molecular Devices, Sunnyvale, CA, USA).

### 2.5. LDH Assay

MCF-7 and MDA-MB-231 human breast cancer cell lines were seeded into a 96-well plate with growth medium and grown for 24 h, followed by treatment with JI017 for 24 h. LDH cytotoxicity assay (LDH cytotoxicity assay) was performed according to the manufacturer’s protocols. The fluorescence was determined by measuring the absorbance of the samples at 490 or 492 nm using the ELISA reader (SpectraMax190, Microplate Reader, Molecular Devices, Sunnyvale, CA, USA).

### 2.6. Caspase-3 Activity Assays

MCF-7 and MDA-MB-231 human breast cancer cell lines were seeded into a 6-well plate with growth medium and grown for 24 h. Caspase-3 activity assay (the Biovision colorimetric caspase-3 assay kit) was performed according to the manufacturer’s protocols. The fluorescence was analyzed at 405 nm using a spectrophotometer (Molecular Devices, San Jose, CA, USA).

### 2.7. Intracellular Ca^2+^ Assays

MCF-7 and MDA-MB-231 human breast cancer cell lines were seeded into a 96-well plate with growth medium and grown for 24 h, Then, the cells were treated with JI017. Intracellular calcium assays were performed using a Ca^2+^ Assay Kit (Colorimetric) (Abcam; ab102505), as described in the supplier’s manual. The fluorescence was measured and analyzed at 575 nm using a microplate reader (Molecular Devices, San Jose, CA, USA).

### 2.8. Intracellular ROS Assays

MCF-7 and MDA-MB-231 human breast cancer cell lines were seeded and incubated into a 96-well plate with growth medium and grown for 24 h, followed by treatment with JI017. After the treatment, cells were incubated with the cell permeant 2′7′-dichlorodihydrofluorescein diacetate (CM-H_2_DCFDA, Invitrogen, Waltham, MA, USA) for 30 min at 37 °C, as described in the supplier’s protocol. The fluorescence was measured and analyzed at 495 nm (Ex)/525 nm (Em) using a microplate reader (Molecular Devices, San Jose, CA, USA).

### 2.9. Establishment of Paclitaxel-Resistant MCF-7 and MDA-MB-231 Cell Lines

MCF-7 and MDA-MB-231 human breast cancer cell lines were seeded into 60 mm dishes and exposed to increasing doses of paclitaxel for 6 months. To establish paclitaxel-resistant cell lines, MCF-7R and MDA-MB-231R, paclitaxel (2 nM, half of the IC_50_) treatment was initiated and gradually increased to 100 nM over a period of 6 months.

### 2.10. Colony Formation Assay

Human breast cancer cells and paclitaxel resistant human breast cancer cells (MCF-7, MCF-7R, MDA-MB-231, and MDA-MB-231R) were seeded in 60 mm dishes with growth medium and grown for 24 h at 37 °C in a CO_2_ incubator. Cells were incubated for 10 days for colony formation, and then the colonies were stained with 0.5% crystal violet (Amresco, Solon, OH, USA). To calculate the survival fraction, the number of colonies formed was divided by the number of seeded colonies formed on the control plate.

### 2.11. Transfection

MCF-7 and MDA-MB-231 human breast cancer cell lines were transfected with double-stranded siRNAs (30 nmol/mL) for GRP78 (Santacruz), PERK (Santacruz), CHOP (Bioneer), and Nox4 (Santacruz) in a 6-well plate for 24 h using Lipofectamine 2000 reagent (Invitrogen) according to the manufacturer’s protocol.

### 2.12. Isolation of Total RNA and Protein

Total RNA (10 µg) from human breast cancer cell lines MCF-7 and MDA-MB-231 in a 100 mm cell culture dish was prepared using TRIzol reagent according to the manufacturer’s instructions (Invitrogen). Protein (15 µg) from the cell lysates was collected using the radioimmunoprecipitation assay lysis buffer (Bio-Rad, Hercules, CA, USA). The supernatant was analyzed to quantify the collected protein using the bicinchoninic acid (BCA) method (Thermo Scientific, Rockford, Waltham, MA, USA).

### 2.13. Real-Time PCR and Western Blot Analyses

Real-time (RT) PCR was performed in triplicate using an ABI Power SYBR green PCR Master Mix (Applied Biosystems, Waltham, MA, USA) with E-cadherin specific primers [5′-GAACGCATTGCCACATACAC-3′ (sense) and 5′-GAATTCGGGCTTGTTGTCAT-3′ (antisense)] (NM_004360.5), HIF-1α specific primers [5′-CATAAAGTCTGCAACATGGAAGGT-3′ (sense) and 5′-ATTTGATGGGTGAGGAATGGGTT-3′ (antisense)] (NM_001530.4), vimentin specific primers [5′-GAGAACTTTGCCGTTGAAGC-3′ (sense) and 5′-GCTTCCTGTAGGTGGCAATC-3′ (antisense)] (NM_003380.5), ATF4 specific primers [(5′-AAGCCTAGGTCTCTTAGATG-3′ (sense) and 5′-TTCCAGGTCATCTATACCCA-3′ (antisense)] (NM_001675.4), and CHOP specific primers [(5′-ATGAGGACCTGCAAGAGGTCC-3′ (sense) and 5′-TCCTCCTCAGTCAGCCAAGC-3′ (antisense)] (NM_001195053.1) on a Roche LightCycler 96 System (Roche). RNA quantities were normalized using β-actin primers [5′-AAGGCCAAC CGCGAGAAGAT-3′ (sense) and 5′-TGATGACCTGGCCGTCAGG-3′ (antisense)], and gene expression analyses was quantified according to the 2^−ΔCt^ method. Real-time PCR was conducted by subjecting the samples to 40 cycles of 94 °C for 30 s, 58 °C for 1 min and 72 °C for 30 s. In Western blot analysis, cells were solubilized in RIPA lysis buffer (Bio-Rad). Then, blocked membranes were incubated with primary antibodies at 4 °C overnight. The primary antibodies used included β-actin (Santa Cruz, 1:1000, sc-47778), eIF2α (Santa Cruz, 1:1000, sc-133132), GRP78 (Santa Cruz, 1:1000, sc-166490), CD63 (Abcam, 1:1000, ab216130), Nox4 (Proteintech, 1:1000, 14347-1-AP), cleaved caspase-3 (Cell Signaling, Danvers, 1:1000, #9664), cleaved caspase-9 (Cell Signaling, 1:1000, #20750), p-PERK(Thr980) (Cell Signaling, 1:1000, #3179), PERK (Cell Signaling, 1:1000, #5683), p-eIF2α (Ser51) (Cell Signaling, 1:1000, #3398), ATF4 (Cell Signaling, 1:1000, #11815), CHOP (Cell Signaling, 1:1000, #2895), E-cadherin (Cell Signaling, 1:1000, #14472), HIF-1α (Cell Signaling, 1:1000, #13116), Slug (Cell Signaling, 1:1000, #9585), Snail (Cell Signaling, 1:1000, #3879), and vimentin (CellSignaling, 1:1000, #5741). After membrane washing, it was incubated for 40 min at room temperature with a 1:4000 diluted HRP-conjugated secondary antibodies. The secondary antibodies used included anti-mouse anti rabbit IgG HRP-linked antibody (Santa Cruz, sc-2357) and m-IgGK BP-HRP-linked antibody (Santa Cruz, sc-516102). The membranes were analyzed by ECL Prime Western Blotting Detection Reagents (Amersham, UK).

### 2.14. Exosome Isolation

MCF-7 and MDA-MB-231 human breast cancer cell lines were treated with JI017 at the doses shown, and exosomes were obtained from the supernatant of JI017-treated MCF-7 and MDA-MB-231 cells according to the manufacturer’s protocol (Total Exosome Isolation Reagent (for cell culture media), Thermo Fisher Scientific, Rockford). Protein concentration was measured using the BCA method (Thermo Scientific). The protein loading samples (10 μg) were also quantified by Ponceau S staining and subjected to Western blot analysis. Positive exosomes were identified using the exosome marker CD63.

### 2.15. Animals

For animal study, 5-week-old, female, athymic BALB/c nude mice (*nu*/*nu*) were purchased from OrientBio, Inc. (Daejeon, Korea), and maintained for 1 week with ad libitum access to sterile standard mouse chow (NIH-7 open formula) and water before use. Mice were housed randomly at 50 ± 20% humidity and approximately 21 ± 2 °C on a 12 h light–dark cycle (*n* = 10 mice/group). All animal experimental procedures were performed according to the National Institutes of Health guidelines and a protocol approved by the Institutional Animal Care and Use Committee of Kyung Hee University.

### 2.16. Tumor Xenograft Mouse Models

For mice xenograft experiment, mice, aged 6 weeks, were inoculated with MDA-MB-231 human breast cancer cell line by subcutaneously (sc) implanting 1 × 10^7^ cultured cells into the right thigh. Mice were grouped randomly (*n* = 10 per group) 6 day later, and JI017 (400 or 600 mg/kg) was administered intraperitoneally (ip) once a day for 2 days. Tumor sizes on two axes (*L*, longest axis; *W*, shortest axis) were measured three times per week using Vernier calipers. Tumor volume was calculated as (*L* × *W*^2^)/2 (mm^3^).

### 2.17. Statistical Analysis

All results were confirmed using at least three independent experiments. Student’s *t*-test was used to compare the means of quantitative data between groups, and a *p* value < 0.05 was considered statistically significant.

## 3. Results

### 3.1. Anticancer Effects of JI017 In Vitro and In Vivo in Breast Cancer Cells

**Figure 1:**
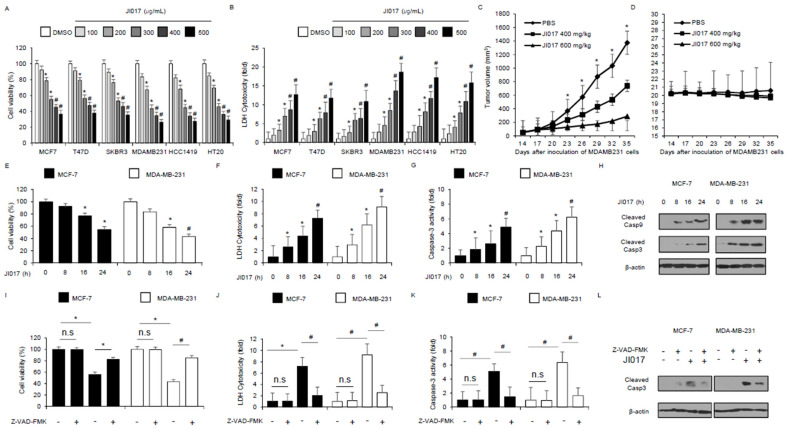
Cytotoxic effects of JI017 in breast cancer cell lines. (**A**,**B**) Cell viability and LDH cytotoxicity of a dose-dependent JI017, in breast cancer cell lines, including MCF-7, T47D, SK-BR-3, MDA-MB-231, HCC-1419, and HT20, measured using the WST-1 assay and LDH assay on 96-well plates; *, *p* < 0.05, #, *p* < 0.01. (**C**,**D**) MDA-MB-231 cells (1 × 10^7^) were implanted (sc) into the thigh on the right hind leg of nude mice (*n* = 10/group). JI017 (400 or 600 mg/kg) or PBS was administered (ip) once a day for two days. The longest (L) and shortest (W) axes of the tumors were measured, and the tumor volume (mm^3^) was calculated as LW2/2. Body weights of the MDA-MB-231 tumor-xenograft mice were determined twice a week during the experiment. (**E**–**H**) JI017 is treated in a time-dependent manner (0, 8, 16, and 24 h; 300 µg/mL) and the WST-1 assay, LDH assay, and caspase-3 activity assays were performed. Western blotting analyses of cleaved caspase-3 and -9 for the indicated times in JI017-treated MCF-7 and MDA-MB-231 cells; *, *p* < 0.05, #, *p* < 0.01. β-actin was used as a protein loading control. (**I**–**L**) The effect of Z-VAD-FMK (50 μM) and JI017 treatment. MCF-7 and MDA-MB-231 cells were pre-treated with Z-VAD-FMK for 4 h and were subsequently treated with JI017 (300 µg/mL, 24 h). Cell viability was determined using the WST-1 assay; cell cytotoxicity was monitored using the LDH assay, and caspase-3 activity was assessed using the caspase-3 activity assay; *, *p* < 0.05, #, *p* < 0.01. To identify caspase-3 cleavage, Western blot assay performed. β-actin was used as a protein loading control.

To study the anti-cancer effect of JI017 in breast cancer cell lines, including MCF-7, T-47D, SK-BR-3, MDAM-MB-231, HCC-1419, and HT-20, we tested the cell viability and LDH cytotoxicity using WST-1 and LDH assays, respectively, in a dose-dependent manner (Figure 1A,B). Compared with the control treatment, which involved DMSO, JI017 treatment resulted in reduced cell viability and increased LDH cytotoxicity in a dose-dependent manner in breast cancer cell lines (Figure 1A,B). To identify the effects of JI017 in vivo, a breast cancer xenograft mice model was constructed with MDA-MB231 cells. Mice in the 400 mg/kg and 600 mg/kg JI017 groups showed lower tumor volumes than the control group and exhibited dose-dependent efficacy (Figure 1C). The body weight did not significantly differ among the groups (Figure 1D). To confirm the anticancer effect of JI017, we tested the cell viability, cell cytotoxicity, and caspase-3 activity using WST-1, LDH cytotoxicity, and caspase-3 activity assays, respectively, in a time-dependent manner (Figure 1E–G). JI017 treatment induces the reduction in cell viability and increase in LDH production, and caspase-3 activity in a time-dependent manner in the breast cancer cell lines MCF-7 and MDA-MB-231 (Figure 1E–G). On performing Western blot, we found that JI017 treatment mediates caspase-3 and -9 cleavage in a time-dependent manner (Figure 1H). To study whether JI017 regulates caspase-dependent apoptosis in breast cancer cells, we performed a pharmacological inhibitor experiment using caspase inhibitor Z-VAD-FMK. Z-VAD-FMK alone did not change cell viability, LDH cytotoxicity, and caspase-3 activity; however, JI017 alone reduced cell viability and increased LDH production, and caspase-3 activity. JI017 in combination with Z-VAD-FMK markedly inhibited the decrease in cell viability and increase in LDH cytotoxicity and caspase-3 activity (Figure 1I–K). Furthermore, Western blot analyses indicated that the combination of JI017 and Z-VAD-FMK blocked caspase-3 cleavage to a greater extent than JI017 alone (Figure 1L). These results suggested that JI017 treatment induces apoptosis in breast cancer cells.

### 3.2. JI017 Treatment Mediates ER Stress Response in Breast Cancer Cells

**Figure 2:**
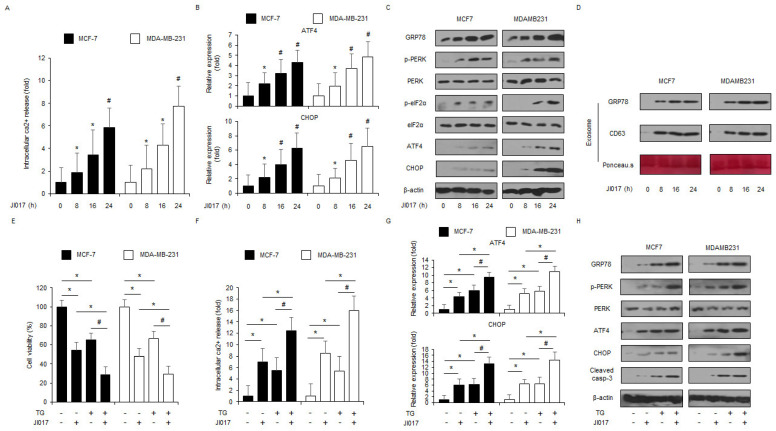
JI017 induces ER stress and cell death via intracellular Ca^2+^ release. (**A**) MCF-7 and MDA-MB-231 cells were treated with JI017 (300 µg/mL) in a time-dependent manner (0, 8, 16, and 24 h) and intracellular Ca^2+^ release was determined using intracellular Ca^2+^ assay; *, *p* < 0.05, #, *p* < 0.01. (**B**) MCF-7 and MDA-MB-231 were treated with JI017 (300 µg/mL) in a time-dependent manner (0, 8, 16, and 24 h) and mRNA levels of ATF4 and CHOP were investigated using real-time RT-PCR; *, *p* < 0.05, #, *p* < 0.01. (**C**) MCF-7 and MDA-MB-231 cells were treated with JI017 (300 µg/mL) for the indicated times (0, 8, 16, and 24 h) and the activation of ER stress signaling, including GRP78, p-PERK, PERK, p-eIF2α, eIF2α, ATF4, and CHOP, was assessed using the Western blot assay. β-actin was used as a protein loading control. (**D**) MCF-7 and MDA-MB-231 cells were treated with JI017 (300 µg/mL) for the indicated times (0, 8, 16, and 24 h) and exosomes were extracted from the cell culture media. Protein samples extracted from cell lysates and exosomes was quantified by Ponceau S staining. These samples were conducted by Western blotting using the ER stress marker, GRP78 and the exosome marker, CD63. (**E**–**H**) Real-time RT-PCR of ATF4 and CHOP and Western blotting of GRP78, p-PERK, PERK, ATF4, CHOP, and cleaved caspase-3 levels were determined using WST-1 assay and intracellular Ca^2+^ assay in thapsigargin (TG; 3 μM, 24 h) and JI017 (300 µg/mL, 24 h)-treated breast cancer cells; *, *p* < 0.05, #, *p* < 0.01.

Ca^2+^ is a potential regulator of cell death and cell survival, and it induces apoptosis by regulating the early and late stages of apoptosis, UPR, and protein folding in various conditions [34]. To identify if JI017 regulates Ca^2+^ production, intracellular Ca^2+^ assay was performed. When MCF-7 and MDA-MB-231 cells were treated with JI017, they showed approximately 6–8 fold higher intracellular Ca^2+^ release than the controls in a time-dependent manner (Figure 2A). We performed RT-PCR to study the mRNA expression of ER stress-related genes, such as *ATF4* and *CHOP*, in JI017-treated MCF-7 and MDA-MB-231 cells in a time-dependent manner. JI017-treated cells showed upregulated *ATF4* and *CHOP* when comparison compared with control cells (Figure 2B). We performed Western blot analyses to confirm the protein expression of ER stress markers, including GRP78, p-PERK, PERK, p-eIF2α, eIF2α, ATF4, and CHOP, in JI017-treated MCF-7 and MDA-MB-231 cells in a time-dependent manner. JI017-treated cells showed higher expression levels of GRP78, p-PERK, p-eIF2α, ATF4, and CHOP than control cells (Figure 2C). Many reports suggest that GRP78 regulates cell survival and cell death via cell–cell communication in exosome isolates. To study if JI017 treatment mediates GRP78 in MCF-7 and MDA-MB-231 exosome fractions, we isolated the exosome from the culture medium of JI017-treated breast cancer cells and performed Western blot analyses for JI017-treated exosomal proteins. JI017 treatment upregulated exosome GRP78 and exosome marker CD63 in a time-dependent manner (Figure 2D). To determine if JI017 treatment induces apoptosis via ER stress response, we co-treated the cells with JI017 and the ER stress inducer TG. TG in combination with JI017 reduced cell viability and enhanced Ca^2+^ production to a greater extent than JI017 or TG alone (Figure 2E,F). Combination treatment with TG and JI017 mediated the increase in ATF4 and CHOP mRNA levels and GRP78, p-PERK, ATF4, CHOP, and caspase-3 cleavage protein levels in MCF-7 and MDA-MB-231 cells to a greater extent than TG or JI017 alone (Figure 2G,H). Our findings indicated that JI017 treatment mediates apoptosis through the PERK-ATF4-CHOP signaling pathway in breast cancer cells.

### 3.3. Targeting ER Stress Suppresses JI017-Mediated Cell Death in Breast Cancer Cells

**Figure 3:**
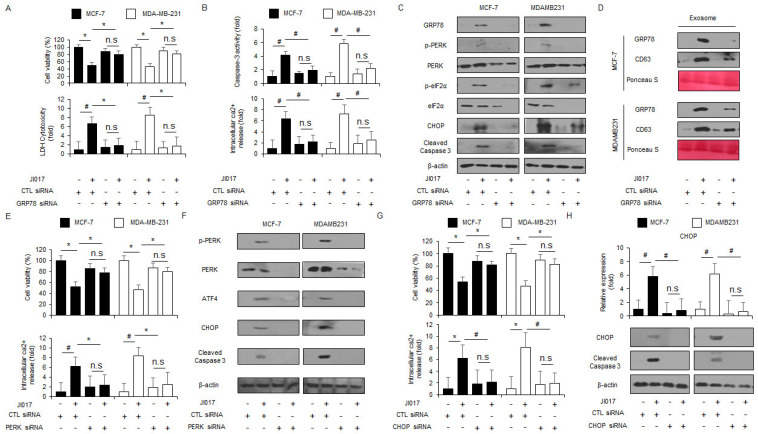
Targeting of ER stress signaling suppresses JI017-induced ER stress and cell death in breast cancer cells. (**A**,**B**) MCF-7 and MDA-MB-231 cells were transfected with GRP78-specific siRNA in the presence or absence of JI017 (300 µg/mL, 24 h) and WST-1, LDH, caspase-3 activity, and intracellular Ca^2+^ assay were performed; *, *p* < 0.05, #, *p* < 0.01. (**C**) Western blot analysis of GRP78, p-PERK, PERK, p-eIF2α, eIF2α, ATF4, CHOP, and cleaved caspase-3 in JI017 (300 µg/mL, 24 h)-treated MCF-7 and MDA-MB-231 cells in the presence or absence of GRP78 siRNA (30 nM, 24 h). β-actin was used as protein loading controls. (**D**) Western blot analysis of GRP78 and CD63 in exosomes extracted from JI017 (300 µg/mL, 24 h)-MCF-7 and MDA-MB-231 cell culture media in the presence or absence of GRP78 siRNA (30 nM, 24 h). β-actin and Ponceau S were used as protein loading controls. (**E**,**F**) After MCF-7 and MDA-MB-231 cells were transfected with PERK (30 nM, 24 h), WST-1, LDH cytotoxicity assay, and Western blotting analyses were performed with/without JI017 (300 µg/mL, 24 h) treatment.; *, *p* < 0.05, #, *p* < 0.01. Western blotting was performed to identify the ER stress-related genes p-PERK, PERK, ATF4, CHOP, and caspase-3 cleavage in JI017-treated PERK knockdown cells. β-actin was used as a protein loading control. (**G**,**H**) After MCF-7 and MDA-MB-231 cells were transfected with CHOP (30 nM, 24 h), WST-1, LDH cytotoxicity assay, Real-time RT-PCR, and Western blotting analyses were performed with/without JI017 (300 µg/mL, 24 h) treatment.; *, *p* < 0.05, #, *p* < 0.01. Real-time RT-PCR was performed to identify the expression of CHOP and Western blotting was performed to identify the expression of CHOP and caspase-3 cleavage in JI017-treated CHOP knockdown cells. β-actin was used as a protein loading control.

To confirm whether JI017 treatment regulates the ER stress master regulator GRP78, GRP78-specific siRNAs were transfected into MCF-7 and MDA-MB-231 cells, which were then treated with JI017. In a knockdown experiment, GRP78 siRNAs blocked the decrease in cell viability and increase in LDH production, Ca^2+^ production, and caspase-3 activity to a greater extent in JI017-treated MCF-7 and MDA-MB-231 cells (Figure 3A,B). Western blot analyses revealed that GRP78 inhibition downregulated GRP78, p-PERK, PERK, p-eIF2α, eIF2α, CHOP, and caspase-3 cleavage levels in JI017-treated MCF-7 and MDA-MB-231 cells to a greater extent (Figure 3C). Furthermore, in exosomes isolated from JI017-treated MCF-7 and MDA-MB-231 cells, GRP78 knockdown downregulated the expression levels of exosome GRP78 in JI017-treated MCF-7 and MDA-MB-231 cells (Figure 3D). Our findings suggest that the inhibition of GRP78 suppresses ER stress and apoptosis in JI017-treated breast cancer cells. To check whether JI017 treatment regulates ER stress and cell death, PERK- and CHOP-specific siRNAs were transfected into MCF-7 and MDA-MB-231 cells, and these cells were then treated with JI017. Unlike transfection of control siRNA, transfection of PERK and CHOP siRNAs blocked the decrease in cell viability and increase inCa^2+^ production in JI017-treated MCF-7 and MDA-MB-231 cells (Figure 3E,G). Western blot analyses revealed that PERK and CHOP knockdown reduced p-PERK, PERK, ATF4, CHOP, and caspase-3 cleavage levels in JI017-treated MCF-7 and MDA-MB-231 cells to a greater extent (Figure 3F,H). In real-time RT-PCR, CHOP knockdown decreased CHOP levels in JI017-treated MCF-7 and MDA-MB-231 cells to a greater (Figure 3H). Our findings suggest that targeting PERK and CHOP blocks ER stress and apoptosis by JI017 treatment in breast cancer cells.

### 3.4. JI017 Treatment Mediates ER Stress and Cell Death by Releasing ROS in Breast Cancer Cells

**Figure 4:**
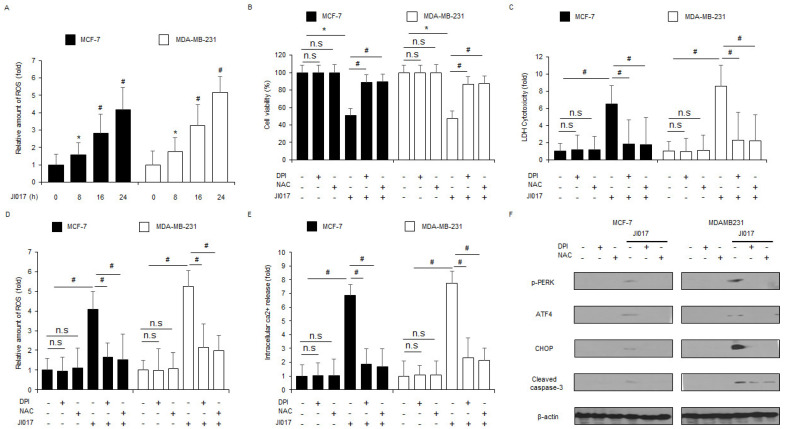
ROS inhibition by DPI or NAC blocks JI017-induced ER stress and cell death in breast cancer cells. (**A**) The fluorescence data indicates intracellular ROS production by DCFDA dye in JI017 (0, 8, 16, and 24 h; 300 µg/mL)-treated MCF-7 and MDA-MB-231 cells; *, *p* < 0.05, #, *p* < 0.01. (**B**–**F**) MCF-7 and MDA-MB-231 cells were pretreated with DPI (1 μM) and NAC (100 μM) for 4 h and subsequently treated with JI017 (300 µg/mL, 24 h). Then, WST-1, LDH cytotoxicity assay, ROS assay, intracellular Ca^2+^ assay, and Western blotting analyses were performed; *, *p* < 0.05, #, *p* < 0.01. (**F**) Western blot showing p-PERK, ATF4, CHOP, and cleaved caspase-3 levels was performed using these samples. β-actin was used as the protein loading control.

To confirm if JI017 treatment regulates ROS release in breast cancer cells, intracellular ROS assay was performed. JI017 induces intracellular ROS release in a time-dependent manner (Figure 4A). To study if ROS inhibitors DPI and NAC suppress JI017-treated ROS release and apoptosis in MCF-7 and MDA-MB-231 cells, we performed the WST-1 assay, LDH assay, intracellular ROS assay, and intracellular Ca^2+^ assay. The combination of JI017 with DPI and with NAC inhibited the decrease in cell viability and increase in LDH release, ROS production, and Ca^2+^ release to a greater extent than JI017 treatment alone (Figure 4B–E). Western blot analyses revealed that co-treatment of JI017 with DPI or NAC blocked p-PERK, ATF4, CHOP, and caspase-3 cleavage expression to a greater extent than JI017 treatment alone (Figure 4F). Our findings indicate that J017 treatment mediates ER stress and apoptosis by producing ROS in breast cancer cells.

### 3.5. JI017 Induces ER Stress and Apoptosis via Nox4 and ROS Release in Breast Cancer Cells

**Figure 5:**
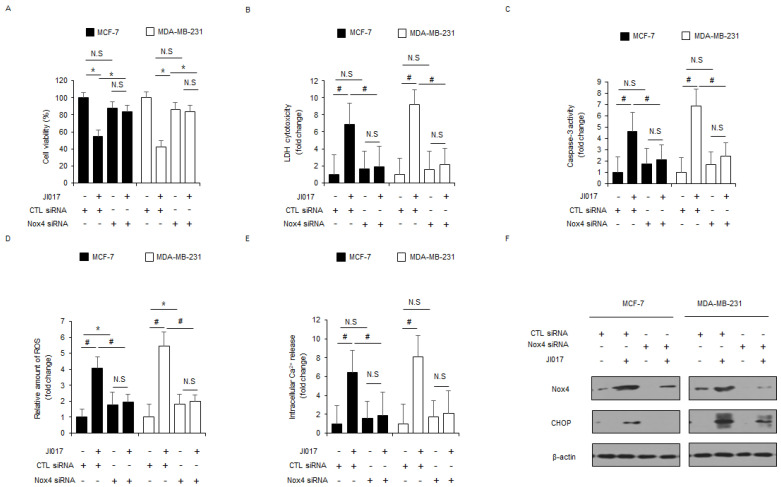
Suppression of Nox4 inhibits JI017-caused ER stress and cell death in breast cancer cells. (**A**–**F**) MCF-7 and MDA-MB-231 cells were transfected with Nox4 siRNAs and treated with JI017 (300 µg/mL, 24 h). Next, WST-1 assay, LDH cytotoxicity assay, caspase-3 activity assay, intracellular ROS assay, and intracellular Ca^2+^ assay were performed along with Western blot analysis for Nox4 and CHOP; *, *p* < 0.05, #, *p* < 0.01. β-actin was used as the protein loading control.

To identify whether JI017 treatment regulates Nox4 expression and ROS production, Nox4-specific siRNAs were transfected into MCF-7 and MDA-MB-231 cells, and these cells were then treated with JI017. Knockdown experiments of Nox4 indicated higher cell viability and lower LDH release, caspase-3 activity, intracellular ROS release, and intracellular Ca^2+^ release in JI017-treated MCF-7 and MDA-MB-231 cells in comparison to transfection of control siRNA with JI107 treatment (Figure 5A–E). Western blot analyses indicated that Nox4 knockdown decreased Nox4 and CHOP levels to a greater extent in JI017-treated MCF-7 and MDA-MB-231 cells than in control cells (Figure 5F). Our findings suggest that Nox4 is involved in ER stress and apoptotic cell death by ROS production in JI017-treated breast cancer cells.

### 3.6. Paclitaxel in Combination with JI017 Overcome Paclitaxel Resistance by Blocking EMT in Paclitaxel-Resistant Breast Cancer Cells

**Figure 6:**
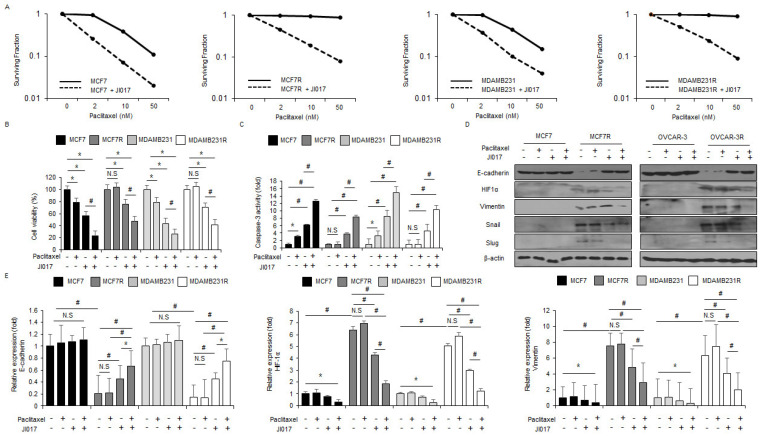
Combined with paclitaxel and JI017 overcome paclitaxel resistance in paclitaxel resistant breast cancer cells. (**A**) A colony formation assay was performed at indicated doses (2, 10, or 50 nM) of paclitaxel and then the survival fraction was calculated using the surviving fraction formula in MCF-7, MCF-7R, MDA-MB-231, and MDA-MB-231R; *, *p* < 0.05, #, *p* < 0.01. (**B**–**E**) MCF-7, MCF-7R, MDA-MB-231, and MDA-MB-231R cells were treated with JI017 (300 µg/mL, 24 h) after exposure to 2 nM paclitaxel. WST-1 assay and caspase-3 activity assay were performed along with a Western blot analysis for E-cadherin, HIF-1α, vimentin, Slug, and Snail and Real-time RT-PCR for E-cadherin, HIF-1α, and vimentin; *, *p* < 0.05, #, *p* < 0.01. β-actin was used as the RNA and protein loading control. β-actin was used as the protein loading control.

To study whether the combination of paclitaxel and JI017 overcomes paclitaxel resistance in breast cancer cells, paclitaxel-resistant MCF-7R and MDA-MB-231R cells were established by exposing MCF-7 and MDA-MB-231 cells to paclitaxel and then performing the colony formation assay, WST-1 assay, LDH assay, Western blot analysis, and RT-PCR. The results indicated that JI017 reduces surviving fraction levels depending on the paclitaxel dose (2, 10, and 50 nM) in MCF-7, MCF-7R, MDA-MB-231, and MDA-MB-231R cells in comparison to control cells (Figure 6A). JI017 reduced cell viability and increased caspase-3 activity in MCF-7 and MDA-MB-231 cells but not in MCF-7R and MDA-MB-231R cells. The combination of paclitaxel and JI017 resulted in mediated lower cell viability and higher caspase-3 activity in MCF-7 and MDA-MB-231 cells than in MCF-7R and MDA-MB-231R cells (Figure 6B,C). To investigate whether the combination of paclitaxel and JI017 blocks EMT in paclitaxel-resistant breast cancer cells, we performed Western blot analyses and RT-PCR. In MCF-7 and MDA-MB-231 cells, JI017 alone, paclitaxel alone, and the combination of paclitaxel and JI017 resulted in no change in the expression levels of EMT-related proteins (Figure 6D,E). However, the level of E-cadherin was downregulated and those of HIF-1α, vimentin, Snail, and Slug were upregulated in MCF-7R and MDA-MB-231R cells (Figure 6D,E). Paclitaxel resulted in almost no expression change; JI017 and the combination of paclitaxel and JI017 in these cells resulted in the upregulation of E-cadherin and downregulation of HIF-1α, vimentin, Snail, and Slug when compared with control cells (Figure 6D,E). Specifically, the combination of paclitaxel and JI017 showed more potent inhibitory effects on EMT than JI017 alone in MCF-7R and MDA-MB-231R cells (Figure 6D,E). Therefore, these results suggest that the combination of paclitaxel and JI017 overcomes paclitaxel resistance via the suppression of EMT in paclitaxel-resistant breast cancer cells.

## 4. Discussion

In this study, we showed that JI017 or paclitaxel with JI017 potently induces ER stress and apoptotic cell death in paclitaxel resistant breast cancer cells and breast cancer cells. In addition, we suggested evidence that sensitivity of paclitaxel resistant breast cancer cells to this combination treatment correlates with EMT process by blocking HIF-1α, vimentin, Snali, and Slug and by activating E-cadherin in paclitaxel resistant breast cancer cells.

EMT phenomenon processed by the loss of epithelial phenotypes and the acquisition of mesenchymal characteristics has been associated with chemoresistance on breast cancer therapy, indicating that inhibition of EMT process represents a rational strategy to overcome chemoresistance [35]. Our finding identified this hypothesis by showing that treatment of paclitaxel resistant MCF-7R and MDA-MB-231R cells with JI017 in combination with paclitaxel decreased cell survival and increased apoptotic cell death in association with the reduction in cell viability and the increase in LDH release. Furthermore, reduced expression of EMT-related genes such as HIF-1α, vimentin, Snail, and Slug and enhanced expression of E-cadherin increased these inhibitory effects of cell survival.

The researchers have reported antitumor effects of herbal medicines such as *Paris polyhylla*, *Saussurea lappa* and *Aucklandia lappa*, *Oryza officinalis*, and *Curcuma longa* via ER stress response [36]. ER stress pathway response regulates ER homeostasis, and it is initiated by three UPR sensors such as PERK, IRE1α, and ATF6 [37]. The PERK/eIF2α/ATF4/CHOP signaling pathway is a potential therapeutic strategy for sensitizing cancer cells [38]. Here, we showed that JI017 induces ER stress in MCF-7 and MDA-MB-231 cells as indicated by the upregulation of exosome GRP78 and cell lysate ER stress markers including GRP78, p-PERK, p-eIF2α, ATF4, and CHOP. These are first report showing the regulation of JI017-induced ER stress response via exosome release. In MCF-7 and MDA-MB-231 cells treated with JI017, the upregulation of exosome marker CD63 and ER stress marker GRP78 mean that JI017 induces ER stress via exosome and cell lysate. More importantly, our study identified that thapsigargin (TG), in combination with JI017, has synergistic anticancer effects in MCF-7 and MDA-MB-231 cells to a greater extent than JI017 or TG alone. TG blocked Ca^2+^ homeostasis via the inhibition of sarco/endoplasmic reticulum (ER) Ca^2+^-ATPase (SERCA) and then induced cell death [39]. The synergistic effect of TG and JI017 co-treatment mean that JI017 also induces ER stress and cell death by inhibiting calcium pump SERCA in breast cancer cells. Moreover, GRP78, PERK, or CHOP knockdown suppressed ER stress and cell death in JI017-treated breast cancer cells, MCF-7 and MDA-MB-231.

ROS exert a potential function to determine cancer cell survival and death and is an important target for anticancer drugs [40,41]. The accumulation of ROS induces by increasing protein-folding in the ER and Nicotinamide adenine dinucleotide phosphate (NADPH) and then it mediates apoptosis via the inhibition of cancer cell growth and thioredoxin reductase, and activation of redox signaling [42,43]. With our results, JI017 induced ER stress and cell death by increasing intracellular ROS production and NADPH oxidase NOX4 activity in MCF-7 and MDA-MB-231 cells. Furthermore, NOX inhibitor DPI and ROS inhibitor NAC blocked ER stress and cell death in JI017-treated MCF-7 and MDA-MB-231 cells. Nox4 binds to p22phox and contributes to cancer development and progression via ROS release and HIF-1α in cancer cells [44].

## 5. Conclusions

In conclusion, we suggest that novel herbal formula JI017 inhibits breast tumor growth via ER stress and cell death and blocks EMT process in paclitaxel resistant breast cancer cells and breast cancer cells. Our results indicate important insights into the molecular mechanism of paclitaxel/JI017 in breast cancer combination therapy.

## Data Availability

All of the data is contained within the article.
